# Comment on “A Rare Case of Renal Infarct due to Noncompaction Cardiomyopathy: A Case Report and Literature Review”

**DOI:** 10.1155/2016/3240695

**Published:** 2016-09-01

**Authors:** Josef Finsterer, Sinda Zarrouk-Mahjoub

**Affiliations:** ^1^Krankenanstalt Rudolfstiftung, 1180 Vienna, Austria; ^2^Genomics Platform, Pasteur Institute of Tunis, 1002 Tunis, Tunisia

With interest we read the article by Wats et al. about a 53-year-old female with left ventricular hypertrabeculation/noncompaction (LVHT) in whom renal infarction was attributed to LVHT [1]. We have the following comments and concerns.

How can the authors be sure that renal infarction originated from LVHT and not from another source [1]? Were atrial fibrillation, heart failure, venous thrombosis, and patent foramen ovale excluded as source of embolism? The patient had an ejection fraction of 25% and dilated cardiomyopathy, conditions which are prone to cardiac embolism. Why did the authors not consider heart failure as the source of embolism? Were aortic plaques, aortic aneurysm, or renal artery stenosis excluded as causes of renal embolism? A further argument against LVHT as the source of embolism is that some studies have shown that the risk of systemic cardioembolism is not increased in patients with LVHT compared to the general population [2].

We do not agree with the statement that LVHT is a genetic disease, due to an arrest in the embryonic compaction process. Though LVHT is associated with mutations in >40 genes [3], none of these mutations have ever been proven to be responsible for LVHT. Arguments against a causal relation between any mutation and LVHT are that these mutations do not segregate with LVHT in most of the families, that it is quite unlikely that the extensive genetic heterogeneity manifests with the same morphology, that most of these mutations manifest with a broad phenotypic heterogeneity, and that most LVHT cases are nonfamiliar. Did any of the first-degree family members of the index case present with LVHT on echocardiography? A further argument against a genetic cause of LVHT is the phenomenon of acquired LVHT, which occurs particularly in patients with neuromuscular disorders (NMDs), in pregnant females, and in professionally training athletes [4]. Was a NMD in the presented patient excluded?

We also do not agree with the statement that Dusek et al., 1975, were the first to describe LVHT. LVHT has been described already in 1969 by Feldt et al. who reported biventricular, bizarre, spongy myocardium in a 3-month-old Caucasian female with complete situs inversus on autopsy [5].

Though the authors mention ventricular arrhythmias and sudden cardiac death extensively in the discussion, they do not mention if the patient had undergone long-term ECG recordings [1]. Did the index patient undergo Holter monitoring and what were the results? Was the family history positive for sudden ventricular arrhythmias, syncopes, or cardiac death? Did any of the first-degree relatives require implantation of an implantable cardioverter defibrillator?

Overall, this interesting case could profit from a discussion about alternative causes of renal infarction, from a more extensive family screening for LVHT, and from a more comprehensive work-up of the patient with regard to source of embolism. To improve the understanding of LVHT, more widespread investigations need to be carried out in individual patients and their relatives.
